# Vena Cava Filter Misplacement: A Killer Traveler

**DOI:** 10.21470/1678-9741-2021-0438

**Published:** 2022

**Authors:** Leonardo Rufino Garcia, André Monti Garzesi, Antonio Sérgio Martins, Marcone Lima Sobreira, Flavio de Souza Brito, Marcello Laneza Felicio

**Affiliations:** 1 Department of Surgery, Universidade Estadual Paulista (UNESP), Botucatu, São Paulo, Brazil.

**Keywords:** Cardiopulmonary Bypass, Thoracic Surgery, Equipment and Supplies, Hemodynamics, Vena Cava Filters

## Abstract

Inferior vena cava filter embolization is not uncommon and can reach 11.8%. However, device migration to the heart is not frequent and occurs in cases after inferior vena cava filter fracture. We present the case of a young woman who was submitted to a routine inferior vena cava filter placement three days before and presented with hemodynamic instability. Since the device was not retrievable, the surgical team opted for an open cardiac surgery under cardiopulmonary bypass to remove the inferior vena cava filter and avoid other further complications.

**Table:** 

Abbreviations, Acronyms & Symbols	
IVCF	= Inferior vena cava filter
VTE	= Venous thromboembolism

## INTRODUCTION

**Fig. 1:**
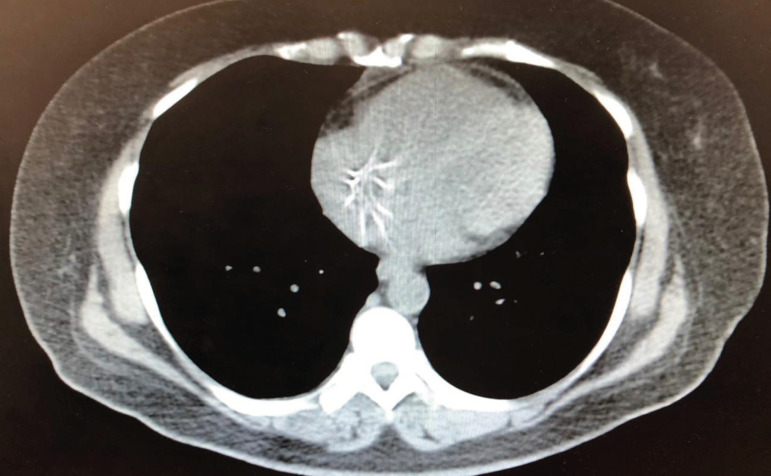
Chest tomography with inferior vena cava filter inside the right atrium.

**Fig. 2:**
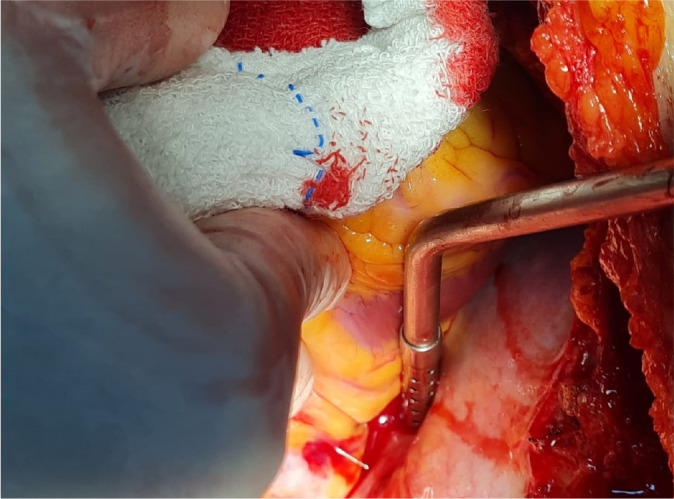
Right atrium perforated by inferior vena cava filter.

In this educational forum, we present the case of a 47-year-old female patient in clinical follow-up because of abnormal uterine bleeding due to uterine myomatosis. Three days after a routine inferior vena cava filter (IVCF) implantation for previous deep vein thrombosis, she presented with tachycardia, hypotension, and hemodynamic instability. The hypothesis of pulmonary thromboembolism was then made. After chest tomography, the medical team verified that the device had migrated to the right atrium (Figure 1), with a punctual perforation diagnosed after pericardiotomy (Figure 2).

## QUESTIONS

- What are the IVCF placement indications?
- What are the possible IVCF complications?
- Which are the IVCF types, and when retrieval is indicated?
- How was the surgical treatment?

### Discussion of Questions

**Question A.** Venous thromboembolism (VTE) is the third most common cause of cardiovascular disease, following myocardial infarction and stroke^[1]^. When it occurs in the lungs as pulmonary thromboembolism, it is associated with a high risk of poor outcomes^[2]^. The first-line therapy for these serious conditions is anticoagulation with heparin products, vitamin K antagonists, and novel oral anticoagulation agents such as apixaban, dabigatran, edoxaban, and rivaroxaban^[3]^.

The absolute indication for IVCF placement is in patients with VTE and contraindication to anticoagulation due to a major risk of bleeding or in patients who have failed this treatment^[1,2]^. Some expanded indications vary according to medical societal guidelines. They include previous anticoagulated patients with recurrent VTE, deep vein thrombosis progression, free-floating proximal or iliocaval deep vein thrombosis, and increased risk of death from secondary embolization^[4]^.

**Question B.** In general, IVCF placement is considered safe, and the mortality rate of this procedure is 0.12%^[5]^. Nevertheless, perioperative and delayed complications might occur (between 5% and 23%)^[6]^ and are more frequent 30 days after placement of not retrieved devices^[1]^. Perioperative complications include access site bleeding, thrombosis, infection, arteriovenous fistula, filter tilt, and incomplete opening. Delayed complications include filter migration, fracture, thrombosis, pulmonary embolism, vessel and organ perforation, and embolization^[2]^.

**Question C.** There are two general types of IVCF. Non-retrievable or permanent — available since the late 1960s and used for patients with long-term contraindication to anticoagulation — and retrievable or temporary — which can be removed after conditions associated with the implant resolves^[1]^. According to a Food and Drug Administration (or FDA) statement in 2010, filter removal is recommended as soon as protection from pulmonary thromboembolism is no longer needed^[2]^.Another indication of filter removal is when there are symptoms from migration or perforation. When IVCF components are outside the vascular system, open surgical procedures take place instead of an endovascular approach^[2]^.

**Fig. 3:**
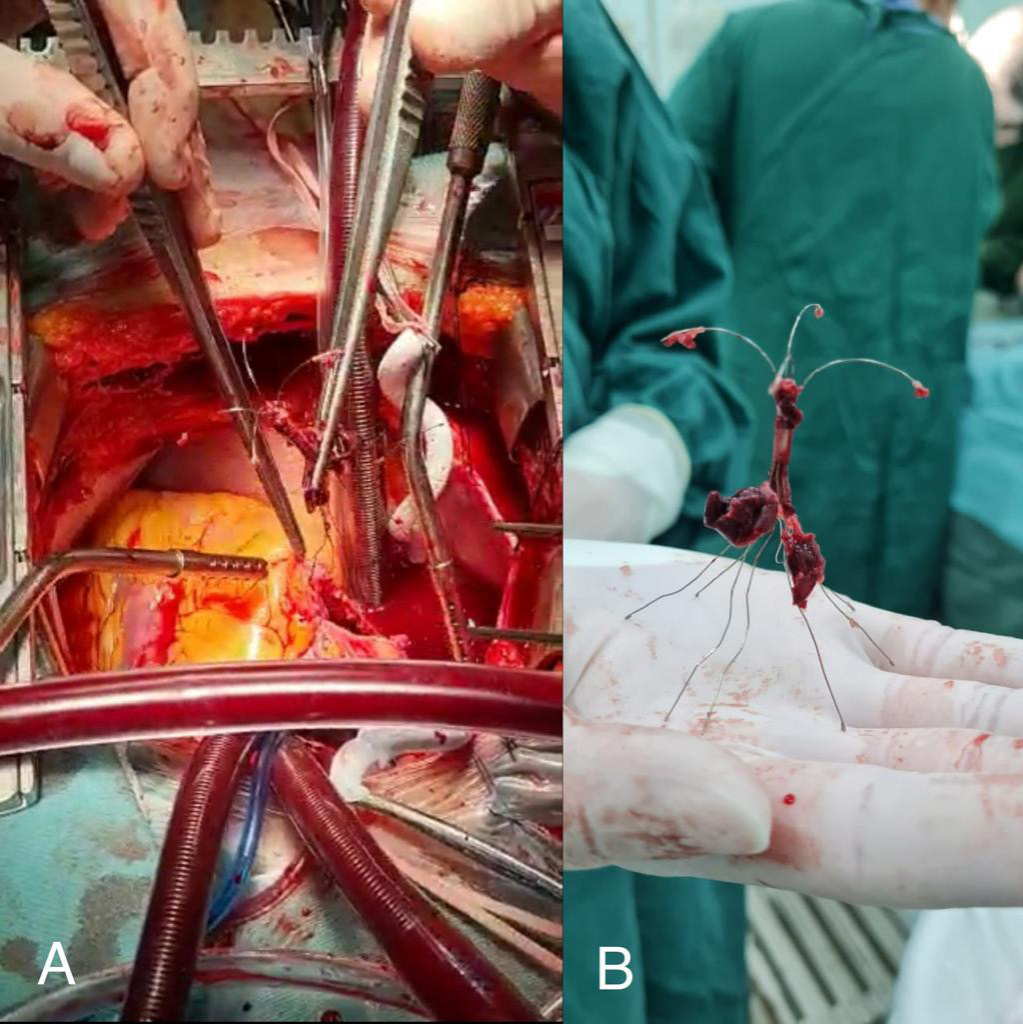
A) Inferior vena cava filter (IVCF) removal after right atriotomy; B) IVCF with clots after removal.

**Question D.** After the clear diagnostic of IVCF embolization to the right atrium (Figure 1), the patient underwent cardiac surgery on an emergency basis. Medium sternotomy, bicaval cannulation, and cardiopulmonary bypass for device resection via right atriotomy were performed. The procedure was carried out in a beating heart fashion with a cardiopulmonary bypass time of 30 minutes. Large clots and the filter were removed (Figure 3A/Video 1). The patient was then weaned off extracorporeal circulation and is currently in postoperative recovery. In Figure 3B we can see IVCF with some clots after removal.

**Video 1:**
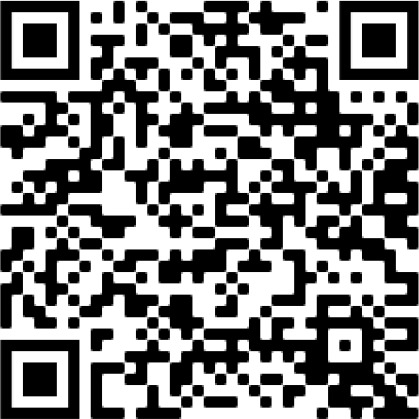
Inferior vena cava filter being removed after cardiopulmonary bypass start via right atriotomy.

## BRIEF CONSIDERATIONS OF THE CASE REPORTED

Filter migration is defined as > 2 cm displacement of the device from its original position, and its rate of occurrence is between 0 and 11.8%^[2]^. Minimal migration may remain asymptomatic. Nevertheless, when it involves the heart, the risk of a potentially fatal complication increases as we can see in the case description. Cardiac surgery teams should be involved in the treatment process since cardiopulmonary bypass techniques are part of their armamentarium.

Filter perforation occurs when there is > 3 mm penetration outside of the vena cava wall. The rate of this complication may vary between 0 and 12.4%. IVCF penetration of adjacent structures can cause important morbidity and mortality. Retroperitoneal and gastrointestinal bleeding, aortic and duodenal penetration, ureter invasion, and cardiac tamponade may happen^[2]^.

## LEARNING POINTS

- Patients with VTE and absolute contraindication to anticoagulation may benefit from IVCF placement^[1,7]^.
- These devices can be permanent or retrievable and complications associated with their placement are well known and more common with not retrieved devices^[8]^.
- In conclusion, assistant physicians and cardiovascular surgeons must keep in mind this potentially fatal complication, particularly when IVCF retrieval is not feasible.
